# A study on promoter characteristics of head-to-head genes in *Saccharomyces cerevisiae*

**DOI:** 10.1186/1471-2164-13-S1-S11

**Published:** 2012-01-17

**Authors:** Darby Tien-Hao Chang, Chi-Yeh Wu, Chen-Yu Fan

**Affiliations:** 1 Department of Electrical Engineering, National Cheng Kung University, Tainan, 70101, Taiwan

## Abstract

**Background:**

Head-to-head (h2h) genes are prone to have association in expression and in functionality and have been shown conserved in evolution. Currently there are many studies on such h2h gene pairs. We found that the previous studies extremely focused on human genome. Furthermore, they only focused on analyses that require only gene or protein sequences but not conducted a systematic investigation on other promoter features such as the binding evidence of specific transcription factors (TFs). This is mainly because of the incomplete resources of higher organisms, though they are relatively of interest, than model organisms such as *Saccharomyces cerevisiae*. The authors of this study recently integrated nine promoter features of 6603 genes of *S. cerevisiae *from six databases and five papers. These resources are suitable to conduct a comprehensive analysis of h2h genes in *S. cerevisiae*.

**Results:**

This study analyzed various promoter features, including transcription boundaries (TSS, 5'UTR and 3'UTR), TATA box, TF binding evidence, TF regulation evidence, DNA bendability and nucleosome occupancy. The expression profiles and gene ontology (GO) annotations were used to measure if two genes are associated. Based on these promoter features, we found that i) the frequency of h2h genes was close to the expectation, namely they were not relatively frequent in genome; ii) the distance between the TSSs of most h2h genes fell into the range of 0-600 bps and was more centralized in 0-200 bps of the highly associated ones; iii) the number of TFs that regulate both h2h genes influenced the co-expression and co-function of the genes, while the number of TFs that bind both h2h genes influenced only the co-expression of the genes; iv) the association of two h2h genes was influenced by the existence of specific TFs such as STP2; v) the association of h2h genes whose bidirectional promoters have no TATA box was slightly higher than those who have TATA boxes; vi) the association of two h2h genes was not influenced by the DNA bendability and nucleosome occupancy.

**Conclusions:**

This study analyzed h2h genes with various promoter features that have not been used in analyzing h2h genes. The results can be applied to other genomes to confirm if the observations of this study are limited to *S. cerevisiae *or universal in most organisms.

## Background

A "head-to-head" (h2h) or "bidirectional" gene pair is a gene organization that two adjacent genes that locate on opposite strands of DNA and transcribe divergently. The "bidirectional promoter" is the inter-genic region between a h2h gene pair [1]. Such an h2h organization has been shown ancient and conserved in evolution [1-3]. Many h2h genes have association in expression and in functionality because that the organization of sharing a bidirectional promoter, which coordinately regulates the transcription of the two h2h genes, makes the related biological process more efficient [1-5].

There have been many studies analyzing the promoter features of h2h genes, including the distance between h2h genes [1,3,4,6], the GC frequency of bidirectional promoter [2,6,7], and the existence of transcription factor binding site (TFBS) [3,7]. However, most of these h2h analyses focused on human genome. Some works [1,3,4,8] compared human h2h genes to those of other organisms such as *fugu*, chicken, mouse and prokaryotes. Nevertheless, their discussions still focused on the conservation of only human h2h genes. In addition to human genome, Gavalas *et al*. [9] and Schuettengruber *et al*. [10] discussed specific h2h genes in chicken and mouse but did not perform a genome-wide analysis. In addition to the organism, the analyzed features in the previous studies were also limited to those requiring only gene or protein (gene product) sequences. This is mainly because that some transcription-related features such as the binding evidence of specific transcription factors (TFs) are more difficult to obtain than gene/protein sequences.

In this study, we conducted several analyses to recognize the characteristics of the bidirectional promoters of associated h2h genes in *Saccharomyces cerevisiae*. The most benefit of using a simple model organism such as *S. cerevisiae *is the considerable resources that are publicly available. Based on the same reason, Wang *et al*. [5] analyzed the h2h genes of *Arabidopsis thaliana *in 2009. For human genome, researchers have to use the existence of TFBS in bidirectional promoters as a compromising way to predict TF-binding. For yeast genome, on the other hand, Monteiro *et al*. have collected 25,180 TF-promoter pairs with experimentally verified binding evidences in 2008 and kept updating their database, YEASTRACT [11]. To date (June 2011), YEASTRACT contained 28,826 TF-binding evidences [12]. The presence or absence of TATA boxes in the promoter is also an important information which has been shown to influence the transcriptional plasticity--the capacity of regulation adjustment upon stimuli [13]. Basehoar *et al*. reported the genomic locations of 2,983 TATA boxes in the promoters of 2,115 yeast genes [8].

Our group recently collected a large amount of promoter features from six databases and five papers, and carefully revised and corrected them into nine kinds of promoter features [14]. These data are valuable to study h2h genes from various features. This study aims to analyze comprehensive features rather than to propose new analyzing algorithms. In this regard, we adopted the established analyzing techniques from previous studies [1,4,5] to examine the expression and functional similarity of 1,504 h2h gene pairs in *S. cerevisiae*. Our results suggest that i) the frequency of h2h genes was close to the expectation, namely they were not relatively frequent in genome; ii) the distance between the TSSs of most h2h genes fell into the range of 0-600 bps and was more centralized in 0-200 bps of the highly associated ones; iii) the number of TFs that regulate both h2h genes influenced the co-expression and co-function of the genes, while the number of TFs that bind both h2h genes influenced only the co-expression of the genes; iv) the association of two h2h genes was influenced by the existence of specific TFs such as STP2; v) the association of h2h genes whose bidirectional promoters have no TATA box was slightly higher than those who have TATA boxes; vi) the association of two h2h genes was not influenced by the DNA bendability and nucleosome occupancy.

These observations expand the knowledge of h2h gene organization. Furthermore, these analyses of h2h genes on various promoter features can be applied to other genomes, of which the results can then been compared with this study to confirm if the observations of this study are limited to *S. cerevisiae *or universal in other organisms.

## Results and discussion

### Identification of h2h gene pairs

We retrieved the genomic locations of the start and stop codons of 6,576 genes from the Saccharomyces Genome Database (SGD) [15] and the transcription start sites (TSSs), 5'UTRs and 3'UTRs of 4,556 genes from [16]. The 6,576 genes form 6,560 pairs of adjacent genes over 16 chromosomes. 626 pairs whose two coding regions are overlapped were excluded. The remaining 5,934 pairs of adjacent non-overlapped genes were categorized into three groups: i) 1,504 h2h gene pairs where the two genes sit on opposite strands and transcribe divergently, ii) 2,856 head-to-tail (h2t) gene pairs where the two genes sit on the same strand and iii) 1,574 tail-to-tail (t2t) gene pairs where the two genes sit on opposite strands and transcribe in a convergent manner. Furthermore, this study created a sub-group of 951 h2h gene pairs where the TSSs of both genes were available. In the following analyses, this sub-group was used if TSS is required; otherwise the three original groups were used.

### Distribution of adjacent genes by chromosome

The distribution of adjacent genes analyzed in this study is shown in Table 1. Though different chromosomes had distinct lengths and number of genes, the gene density was quite stable (5.45 genes per 10 kbp in average). Furthermore, the ratio of h2h gene pairs was stable (25.3% in average) and close to the expectation of 25%. The ratios of h2t and t2t gene pairs were also close to their expectation of 50% and 25%, respectively. Our results indicate that the arrangement of h2h, h2t and t2t in genome is by random, which seems to be conflict to the previous studies [3] claiming that h2h genes are more frequent in genome. Actually in our analysis, t2t should be the most frequent gene organization (26.6% in average) in comparison with the expected frequency.

**Table 1:** Distribution of h2h gene pairs by chromosome

Chr	Length (bp)	#gene	**Density**^1^	#pair	#h2h	#h2t	#t2t	%h2h	%h2t	%t2t
1	230,208	117	5.08	98	26	41	31	26.5	41.8	31.6
2	813,179	456	5.61	412	101	200	111	24.5	48.5	26.9
3	316,617	183	5.78	161	37	82	42	23.0	50.9	26.1
4	1,531,919	837	5.46	757	187	365	205	24.7	48.2	27.1
5	576,869	324	5.62	278	70	134	74	25.2	48.2	26.6
6	270,148	141	5.22	126	32	66	28	25.4	52.4	22.2
7	1,090,947	583	5.34	531	133	264	134	25.0	49.7	25.2
8	562,643	321	5.71	294	74	144	76	25.2	49.0	25.9
9	439,885	241	5.48	217	57	99	61	26.3	45.6	28.1
10	745,741	398	5.34	353	90	165	98	25.5	46.7	27.8
11	666,454	348	5.22	321	81	159	81	25.2	49.5	25.2
12	1,078,175	578	5.36	513	133	237	143	25.9	46.2	27.9
13	924,429	505	5.46	465	122	225	118	26.2	48.4	25.4
14	784,334	435	5.55	397	94	205	98	23.7	51.6	24.7
15	1,091,289	598	5.48	546	145	257	144	26.6	47.1	26.4
16	948,062	511	5.39	465	122	213	130	26.2	45.8	28.0
Overall	12,070,899	6,576	5.45	5,934	1504	2,856	1,574	25.3	48.1	26.5

^1 ^Number of genes per 10 kbp.

This is because that the previous studies used the ratio of genes involved in h2h pairs to all genes. The fact that a gene has two neighbors and involves in two pairs was somehow ignored. We argued that the observation--~50% genes are involved in h2h pairs--in the previous studies is correct, but this number does not indicate that h2h genes enrich in genome. Our analysis, which regarded gene pair a unit and is more accurate in this issue, indicates that the h2h organization is formed nearly by random.

In addition, we propose a bold conjecture that t2t is a gene organization for "storage". More precisely, organisms must store sufficient genes in a limited genome size. However, randomly arranging genes might lead to "interference", co-regulation of two genes that should not be transcribed together. Though organisms have other mechanisms such as microRNA [17] to prevent unwanted transcriptions, arranging them in a t2t manner requires relatively small effort. Based on this conjecture, the higher t2t frequency might be accumulated in evolution where some organisms were extinct because of lethally transcription interference.

### Distance between adjacent genes

**Figure 1:**
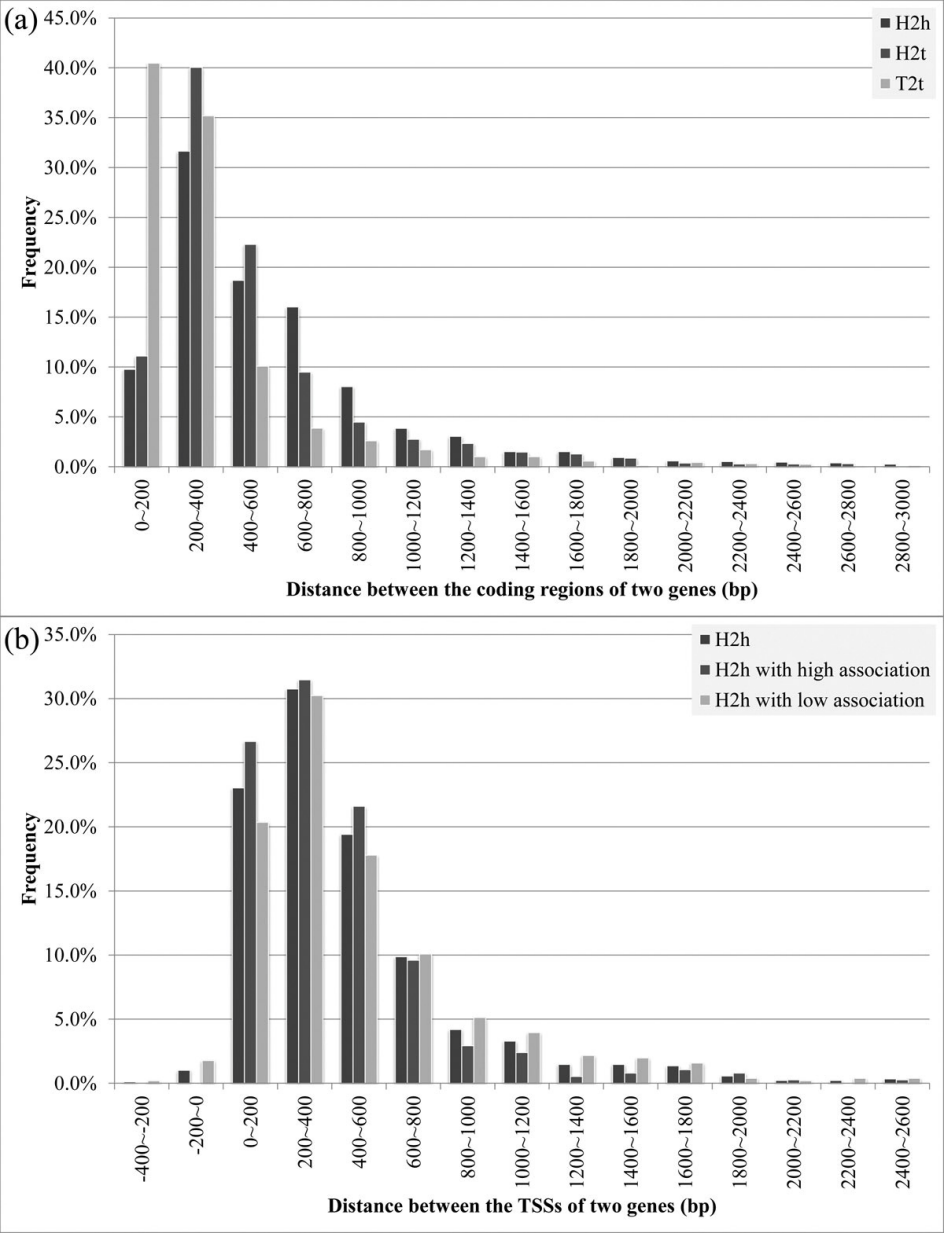
**Distributions of gene distance of adjacent genes.** (a) The gene distance is measured as the number of base pairs in between the coding regions of two genes. (b) The gene distance is measured as the number of base pairs in between the transcription start sites (TSSs) of two h2h genes. H2h with high association represents gene pairs with functional similarity higher than the genome average; while h2h with low association represents the remaining h2h gene pairs (see the Methods section for the details).

The distributions of distance between adjacent genes are shown in Figure 1. Adjacent gene pairs separated by more than 3000 bps, which accounted for less than 3% of adjacent genes, were not shown. Distance between the coding regions, denoted CR distance, of the three kinds--h2h, h2t and t2t--of adjacent gene pairs showed similar distribution plots (Figure 1a), where most gene pairs fell into the range of 0-800 bps (76.1%, 82.9% and 89.6% for h2h, h2t and t2t gene pairs, respectively). We noted that 40.5% t2t gene pairs fell into the range of 0-200 bps, which was obviously higher than h2h (9.8%) and h2t (11.1%) gene pairs. T2t genes with such short CR distances might have overlapped 3'UTRs (the average length of 3'UTRs in our dataset is 147 bps) so that they are unlikely to be transcribed together. This observation reinforces the conjecture that t2t gene is a gene organization for storage, where the phenomenon of many close t2t gene pairs is reasonable for a more compact and efficient storage.

The TSS distances between h2h gene pairs, denoted TSS distance, are shown in Figure 1b. The TSS distances between h2t and t2t gene pairs depend on the lengths of genes' coding regions and were excluded in this analysis. Figure 1b also includes two sub-groups of the h2h genes pairs to observe the TSS differences of h2h gene pairs with different level of functional similarity. In Figure 1b, most h2h gene pairs fell into the range of 0-600 bps (73.2%, 79.7% and 68.4% for h2h, h2h with high association and h2h with low association, respectively). It is noticed that h2h with high association led in all the three bins, while h2h with low association lagged behind other two distributions in all the three bins. In other words, h2h with high association was more "centralized" in the range of 0-600 bps, revealing that the TSS distance is a potential feature to recognize h2h gene association.

Based on this observation, we compared the distribution centrality of CR and TSS distance (H2h in Figure 1a vs. in b). In the TSS distance distribution, the ratio of h2h gene pairs of 0-800 bps was 83.1%, higher than that in the CR distance distribution (76.1%). If we focused on the top three bins, the CR distance distribution (66.4% in 200-800 bps) was also lower than that in the TSS distance distribution (73.2% in 0-600 bps). In this regard, we can say that the distance between TSSs was more centralized, and thus a better feature to recognize h2h gene association than the CR distance.

### Number of TFs in bidirectional promoter

One of the most contributions of this study to previous analyses of h2h genes is using binding and regulation evidence of TFs from literature rather than by prediction. The TF-binding evidence, based on band-shift, footprinting or ChIP assays, indicates whether a TF binds to the promoter of a gene; while the TF-regulation evidence, based on TF knockout assays, indicates whether knocking out a TF leads to a significant change of the expression of a gene. The YEASTRACT database [11,12] has collected the binding and regulation evidences of 28,826 and 19,090 TF-gene pairs, respectively. The YPA database [14] has corrected some of these TF-gene pairs (most of them contained unknown TF or gene identifiers) and resulted in 24,522 TF-binding and 18,871 TF-regulation evidences.

Table 2 shows the co-expression and functional similarity of adjacent genes with different number of common TFs that bind/regulate both genes. The calculation details of co-expression and functional similarity can be found in the Methods section. We found that adjacent genes with the most common TFs show the highest association without depending on the pair type (h2h, h2t or t2t), evidence type (binding or regulation) and evaluation index (co-expression or functional similarity). The number of common TFs is highly correlated to the association of adjacent genes with a Pearson correlation coefficient (PCC) of 0.936 in average, except h2h (PCC=-0.247) and t2t (PCC = 0.396) gene pairs using TF-binding evidence and functional similarity.

**Table 2:** Association of adjacent genes in terms of number of TFs that bind/regulate both genes

		#pair			Co-expression			Functional association		
			
#TF		H2h	H2t	T2t	H2h	H2t	T2t	H2h	H2t	T2t
TFs with binding evidence to both genes										
	0 TF	405	1,761	1,229	0.229	0.221	0.245	1.839	1.641	1.690
	1 TF	301	595	240	0.256	0.215	0.246	1.736	1.727	1.638
	2 TFs	271	246	**105**	0.246	0.229	**0.268**	1.779	1.724	**1.724**
	3 TFs	158	113	**-**	0.261	0.252	**-**	1.663	1.753	**-**
	4 TFs	117	**141**	**-**	0.268	**0.279**	**-**	1.560	**1.971**	**-**
	5 TFs	**252**	**-**	**-**	**0.276**	**-**	**-**	**1.863**	**-**	**-**
	PCC				0.911	0.918	0.899	-0.247	0.877	0.396
TFs with regulation evidence to both genes										
	0 TF	1,053	2,098	1,212	0.232	0.212	0.237	1.673	1.572	1.644
	1 TF	299	522	265	0.260	0.226	0.269	1.885	1.854	1.660
	2 TFs	**152**	**236**	**97**	**0.329**	**0.299**	**0.287**	**2.021**	**2.027**	**2.148**
	PCC				0.972	0.931	0.986	0.992	0.991	0.880
Overall		1,504	2,856	1,574						

Values highlighted with bold font indicate using more than and equal to the number of TFs for sufficient pairs. For example, number of h2h pairs with four TFs with binding evidence is 117; while number of h2t pair with ≥4 TFs with binding evidence is 141. Higher co-expression or functional association indicates better gene association; see the Methods section for the details.

This suggests that more common TFs binding to both promoters of the adjacent genes only strengthened the co-expression but not the functional similarity. On the other hand, more common TFs regulating both adjacent genes strengthened both indices of gene association. This is reasonable since TF-binding evidence does not guarantee the activation of the downstream genes. Here we conclude that the number of TFs regulating both adjacent genes is a critical feature to the association of adjacent genes. However, the TF-regulation evidence (1,053 h2h gene pairs without such information) was rarer than the TF-binding evidence (405 h2h gene pairs without such information) due to the experimental technologies. In this condition, number of TFs binding to both promoters of the adjacent genes is an alternative in applications that require only gene co-expression.

### TFs that prefer regulation of h2h genes

The previous section focuses on the number of TFs in the bidirectional promoter. This section, on the other hand, aims to analyze if there is any specific TF whose existence in the bidirectional promoter determines the association of the h2h genes. We grouped our h2h gene pairs by the common TF. Namely in a group of TF *α*, every pair had *α *as one of its common TFs. Note that pairs with multiple common TFs appeared in multiple groups. We defined the *e-score *and *f-score *of a TF as the average co-expression and functional similarity, respectively, of pairs in the corresponding group. Low e-score/f-score indicates that the h2h genes that the TF binds/regulates have low association. This implies that though the TF binds/regulates both h2h genes, the binding/regulation might be temporally different. Conversely, high e-score/f-score indicates that the TF is prone to bind/regulate the h2h genes in the same time.

Table 3 shows the nine TFs having both high e-scores and f-scores in our dataset. We queried the BioGRID database [18] to check if the h2h gene pairs in the groups of these TFs do have protein-protein interactions (PPIs). The results show that STP2, responding to signals of sensing extracellular amino acids, might be a good indicator of the association of h2h genes--42.9% h2h gene pairs where both genes were bound/regulated by STP2 had PPIs. It also reveals the potential of interactions of the h2h gene pairs having no PPIs reported. Table 4 lists these h2h gene pairs. Similarly, PDR3, activating the pleiotropic drug resistance network, could be another indicator of the association of h2h genes.

**Table 3:** TFs that prefer regulation of h2h genes

TF	**#h2h**^1^	**e-score**^2^	**f-score**^3^	**#PPI**^4^	**%PPI**^5^	**#gene**^6^	**%h2h**^7^
STP2	7	0.375	4.358	3	42.9	235	6.0
RME1	6	0.240	3.437	0	0.0	156	7.7
PDR3	12	0.288	2.931	2	16.7	446	5.4
SFP1	22	0.335	2.452	0	0.0	432	10.2
GCN4	195	0.269	2.250	1	0.5	2,058	19.0
SFP1	42	0.335	2.225	0	0.0	886	9.5
RPN4	41	0.248	2.130	0	0.0	169	48.5
FHL1	57	0.273	2.101	0	0.0	1,120	10.2
MET4	757	0.330	2.034	26	3.4	19,090	7.9

^1 ^Number of h2h gene pairs both bound/regulated by the TF. ^2^ Average co-expression of the h2h gene pairs. ^3^ Average functional similarity of the h2h gene pairs. ^4^ Number of the h2h gene pairs with interactions. ^5^ Ratio of the h2h gene pairs with interactions. ^6^ Number of genes bound/regulated by the TF with evidence. ^7^ Ratio of genes bound/regulated by the TF that are h2h genes.

**Table 4:** H2h pairs where both genes were bound/regulated by STP2

Pair	Systematic name	Gene name	**Evidence**^1^	Gene ontology (GO) annotations
1	YDL234C	GYP7	Regulation	BP GO:0032889 - regulation of vacuole fusion, non-autophagic; CC GO:0005737 - cytoplasm; MF GO:0005097 - Rab GTPase activator activity
	YDL233W	n/a	Regulation	CC GO:0005634 - nucleus
**2**	**YFL060C**	**SNO3**	**Regulation**	**BP GO:0008614 - pyridoxine metabolic process; MF GO:0016740 - transferase activity**
	**YFL059W**	**SNZ3**	**Regulation**	**BP GO:0042823 - pyridoxal phosphate biosynthetic process; MF GO:0005515 - protein binding**
3	YHR136C	SPL2	Regulation	BP GO:0009266 - response to temperature stimulus; CC GO:0005737 - cytoplasm; MF GO:0004860 - protein kinase inhibitor activity
	YHR137W	ARO9	Regulation	BP GO:0009058 - biosynthetic process; CC GO:0005634 - nucleus; MF GO:0016769 - transferase activity, transferring nitrogenous groups
4	YIR027C	DAL1	Regulation	BP GO:0009442 - allantoin assimilation pathway; MF GO:0004038 - allantoinase activity
	YIR028W	DAL4	Regulation	BP GO:0015720 - allantoin transport; CC GO:0016021 - integral to membrane; MF GO:0005274 - allantoin uptake transmembrane transporter activity
**5**	**YMR095C**	**SNO1**	**Regulation**	**BP GO:0008615 - pyridoxine biosynthetic process; CC GO:0005737 - cytoplasm; MF GO:0016740 - transferase activity**
	**YMR096W**	**SNZ1**	**Regulation**	**BP GO:0042823 - pyridoxal phosphate biosynthetic process; MF GO:0005515 - protein binding**
**6**	**YNL334C**	**SNO2**	**Regulation**	**BP GO:0008615 - pyridoxine biosynthetic process; MF GO:0016740 - transferase activity**
	**YNL333W**	**SNZ2**	**Regulation**	**BP GO:0042823 - pyridoxal phosphate biosynthetic process; MF GO:0005515 - protein binding**
7	YOL155C	HPF1	Regulation	BP GO:0031505 - fungal-type cell wall organization; CC GO:0009277 - fungal-type cell wallMF GO:0015926 - glucosidase activity
	YOL154W	ZPS1	Regulation	CC GO:0009277 - fungal-type cell wall

Pairs with known interactions are highlighted with bold font. ^1^ Evidence type (binding or regulation) of STP2 and the gene.

Another aspect of preference is that the TF only binds/regulates h2h genes. The "%h2h" column in Table 3 aims to measure this preference. High ratio indicates the TF usually binds/regulates h2h genes. For example, currently available literature shows that RPN4--stimulating proteasome genes by various stress responses--binds/regulates 169 genes and ~50% of them were h2h genes. However, no TF has both high %PPI and %h2h in Table 3. This suggests that TFs frequently binds/regulates h2h genes are not necessarily to influence the association of the bound/regulated h2h genes.

### Existence of TATA boxes in bidirectional promoter

Many analyses have demonstrated the importance of the presence or absence of TATA boxes in the promoter [13,19]. We obtained the genomic locations of 2,983 TATA boxes from Basehoar *et al*.'s work [8], where 2,022 ones were in the bidirectional promoters of our dataset. Based on these data, the h2h gene pairs were divided into 642 TATA-containing and 862 TATA-less ones according to the presence or absence of TATA boxes in the corresponding bidirectional promoters.

**Figure 2:**
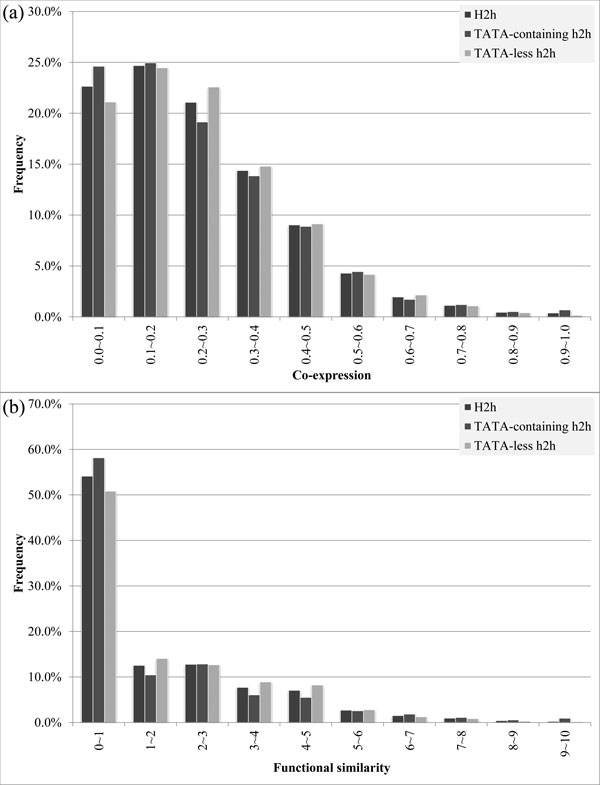
**Gene association of TATA-containing and TATA-less h2h gene pairs.** The ***x***-axis is the co-expression in (a) and functional similarity in (b) while the ***y***-axis is the frequency of h2h gene pairs with the corresponding ***x ***values. Higher ***x ***value indicates better gene association for both indices; see the Methods section for the details.

The distribution plots of co-expression and functional similarity of TATA-containing and TATA-less h2h gene pairs are shown in Figure 2. We found that TATA-containing h2h gene pairs had higher frequency than TATA-less ones in the low co-expression/functional similarity bins and had similar frequency in other bins. H2h gene pairs whose bidirectional promoters containing TATA boxes had slightly lower co-expression (0.239 of TATA-containing vs. 0.246 of TATA-less pairs in average) and functional similarity (1.572 of TATA-containing vs. 1.664 of TATA-less pairs in average). But the small difference between TATA-containing and TATA-less h2h gene pairs reveals that the existence of TATA boxes is not a critical feature to the association of h2h genes.

### Bendability and nucleosome occupancy in bidirectional promoter

The DNA bendability influences the nucleosome positioning and further influences the accessibility of promoter sequences to TFs. TFs have been shown to favor nucleosome-depleted and rigid DNA regions in the promoter [8,13,20]. The YPA database has obtained the nucleosome occupancy at every base pair in the yeast genome from [21] and calculated the bending propensity of each base pair in the yeast genome based on the propensity table of tri-nucleotide in [22]. We used these data to group bidirectional promoters into more and less accessible ones. The bidirectional promoters that are more accessible to TFs were defined as those having at least an accessible sequence segment that is long enough for a TF to bind. In this study, we required that such bidirectional promoters have at least a consecutive sequence segment with ≥8 bps (the average size of TFBSs in the YPA database is 8.634 bps) where every nucleotide has DNA bendability lower than the genome average and has nucleosome occupancy lower than the genome average.

**Figure 3:**
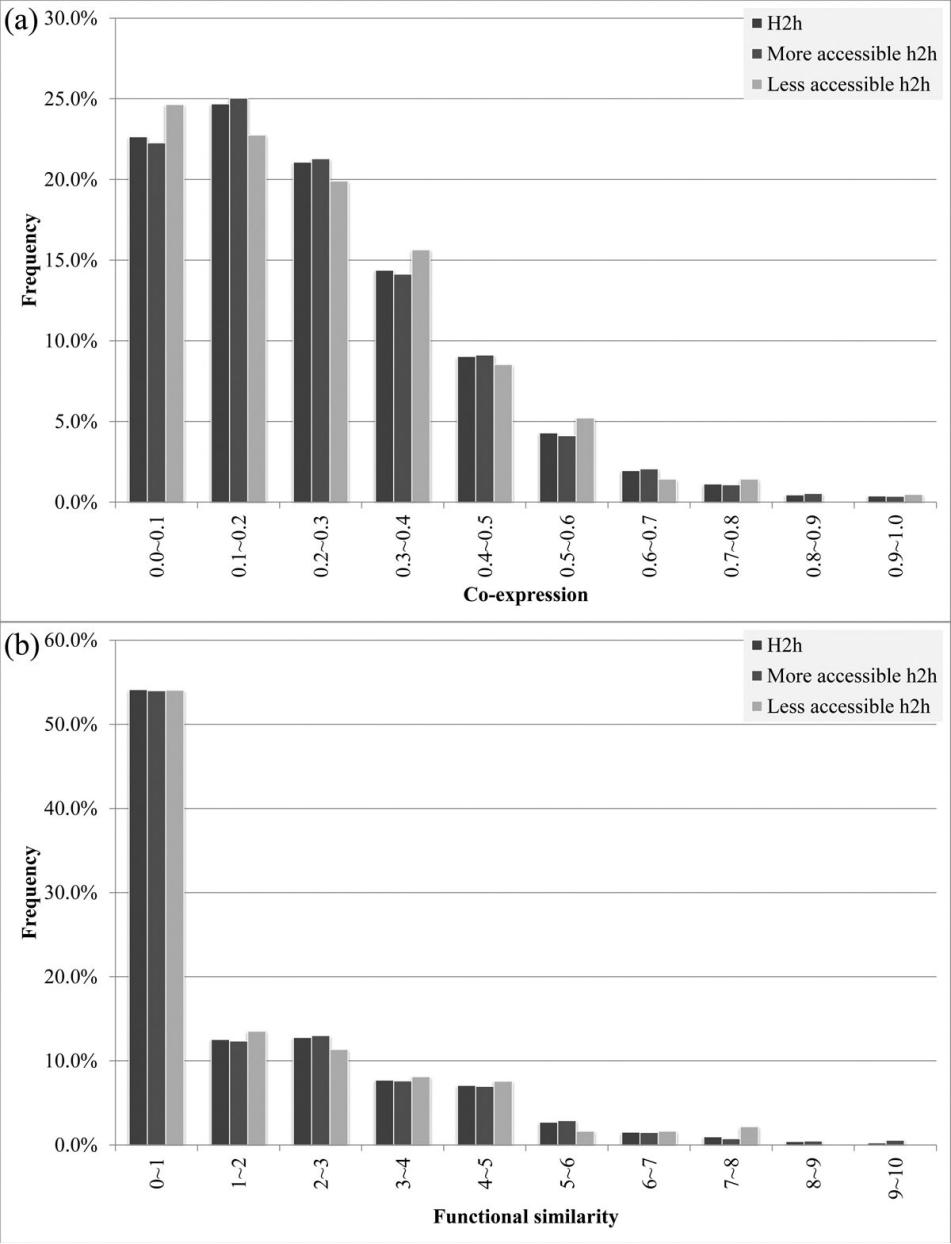
**Gene association of different promoter accessibility.** The ***x***-axis is the co-expression in (a) and functional similarity in (b) while the ***y***-axis is the frequency of h2h gene pairs with the corresponding ***x ***values. Higher ***x ***value indicates better gene association for both indices; see the Methods section for the details.

In Figure 3a, though less accessible h2h gene pairs had higher frequency than more accessible ones in the lowest co-expression bin, they had lower frequency in the next two bins. In general, these three distributions were quite similar. This distribution difference was even smaller in term of functional similarity (Figure 3b). Thus, we conclude that DNA bendability and nucleosome occupancy do not influence the association of h2h genes. This may result from that though nucleosome occupies some promoter regions that are critical to h2h gene regulation, nucleosome will detach from the promoter and spare space for TFs if required.

## Conclusions

A systematic investigation of head-to-head gene organization based on various promoter features was conducted in this work. We echoed and adjusted several known properties of h2h gene organization as well as provided new observations. These analyses can be applied on/compared to h2h genes of other organisms to confirm if the observations of this study are limited to *S. cerevisiae *or universal in most organisms.

## Methods

### Calculation of co-expression

We extracted the expression data of 6,497 genes from 27 microarray datasets collected in the SGD database [15]. After excluding datasets containing less than 15 sample points, we obtained ten microarray datasets which still covered 6,462 genes. For a given gene pair, we calculated their Pearson correlation coefficient (PCC) in each of the ten datasets. The absolute values of these PCCs indicate their co-expression under ten conditions. The datasets not containing the expressions of both genes are ignored. To exclude the possibility that an associated gene pair may only co-express in a certain condition, the highest co-expression, instead of the average co-expression, was used in this study.

### Calculation of functional similarity

We applied the semantic measure in a taxonomy proposed by Resnik [23] on the "biological process" GO subsystem to calculate the functional similarity. We converted the Resnik probability by negative natural logarithm so that higher similarity value indicates better association. The functional similarity of two genes *a *and *b *was defined as follows:

$$Sim\left(a,b\right)=-\ln\left(\frac{\min\left\{gene\left(t_{\text{common}}\right)|\text{both }a\text{ and }b\text{ has }t_{\text{common}}\right\}}{gene\left(t_{\text{root}}\right)}\right),$$

where *gene*(*t*) is the number of genes annotated by GO term *t*; *t*_common _is a common GO term of genes *a *and *b*; *t*_root _is the root GO term; while the fraction within the natural logarithm is the Resnik probability. The phenomenon that *a *and *b *share a GO term of less genes indicates that they have a more specific annotation (less genes have this annotation) in common, thus higher functional similarity. The min {} is used to obtain the most specific common GO term since two genes usually have multiple common GO terms.

## Competing interests

The authors declare that they have no competing interests.

## Authors' contributions

Author DTHC designed the methodology and conceived of this study. CYW and CYF designed the experiments and performed all calculations and analyses. All authors have read and approved this manuscript.
